# A Systematic Review on the Association Between Water Fluoride Levels and Dental Fluorosis: Exploring the ‘Halo Effect’ and Confounding Environmental Factors

**DOI:** 10.3390/ijms27125623

**Published:** 2026-06-22

**Authors:** Mnqweno Funcuza, Bheki T. Magunga, Phoka C. Rathebe, Thokozani P. Mbonane

**Affiliations:** Department of Environmental Health, Faculty of Health Sciences, Doornfontein Campus, University of Johannesburg, Johannesburg 2006, South Africa; bhekim@uj.ac.za (B.T.M.); prathebe@uj.ac.za (P.C.R.)

**Keywords:** dental fluorosis, water fluoridation, halo effect, environmental factors, enamel hypomineralization, systemic fluoride, protease inhibition, altitude, endoplasmic reticulum stress

## Abstract

Dental fluorosis (DF) remains a global public health challenge traditionally attributed to elevated water fluoride F^−^. However, the Halo Effect and environmental factors now complicate this dose–response relationship. Following PRISMA 2020 guidelines, this systematic review identified 20 observational studies (*n* = 21,780) via PubMed, Scopus, and Web of Science. Inclusion logic utilized the PICOS framework, specifically selecting human studies that reported quantitative water F^−^ levels alongside environmental or dietary confounders. Quality was assessed via the Newcastle–Ottawa Scale. Synthesis revealed that in optimal fluoridated areas (0.7 mg/L), mild DF prevalence reached 15–20% in cohorts with high “Halo Effect” exposure (infant formula, processed beverages) a twofold increase over historical benchmarks. High altitude (>2000 m) and arid climates further exacerbated toxicity by altering renal clearance. These factors sustain systemic fluoride levels that inhibit protease activity (MMP-20/KLK4) and induce endoplasmic reticulum stress during enamel maturation, causing hypomineralization. Current water-centric monitoring is insufficient for modern risk assessment. A transition toward Total Daily Intake (TDI) models and context-specific standards accounting for altitude and dietary diffusion is essential to balance caries prevention with systemic safety.

## 1. Introduction

Fluoride (F^−^) is a naturally occurring electronegative ion recognized globally for its role in preventing dental caries. Its therapeutic efficacy is primarily mediated through the promotion of enamel remineralization via the formation of fluorhydroxyapatite and the inhibition of enolase enzymes in cariogenic bacteria, such as Streptococcus mutans [1]. However, the therapeutic window for systemic fluoride intake is narrow. Excessive ingestion during the critical period of odontogenesis, specifically the maturation stage of enamel formation (birth to age eight), can result in Dental Fluorosis (DF) [2]. At the molecular level, DF is characterized by an arrest in the degradation of enamel matrix proteins (EMPs), particularly amelogenins. This biochemical failure leads to increased enamel porosity, reduced mineral density, and the clinical manifestation of opaque white streaks or, in severe cases, confluent pitting and brown staining [3,4]. Current research suggests that fluoride induces this hypomineralization by altering the proteolytic activity of Matrix Metalloproteinase-20 (MMP-20) and Kallikrein-4 (KLK4), as well as by triggering Endoplasmic Reticulum (ER) stress and the Unfolded Protein Response (UPR) in secretory ameloblasts [5]. Historically, the World Health Organization (WHO) established a guideline of 1.5 mg/L in drinking water to mitigate DF risk [6]. Yet, recent epidemiological data indicate a decoupling of water fluoride concentrations from DF prevalence. This shift is largely attributed to the “Halo Effect” (also termed the diffusion effect). Operationally, the Halo Effect is defined as the indirect systemic exposure to fluoride via the consumption of processed foods and beverages that are manufactured in fluoridated regions but distributed to non-fluoridated areas [7]. This phenomenon extends the reach of community water fluoridation (CWF) beyond its geographic point of origin, thereby introducing a hidden variable in the assessment of Total Daily Intake (TDI).

Furthermore, the biological impact of fluoride is not uniform; it is modified by environmental and physiological “potentiators.” Factors such as high altitude have been hypothesized to exacerbate fluoride toxicity through hypoxia-induced metabolic acidosis, while dietary variables, specifically calcium-to-fluoride ratios, modulate the bioavailability of F^−^ in the gastrointestinal tract [8,9]. Despite the documented existence of these variables, existing systematic reviews often focus on localized geographic clusters or overlook the molecular synergy between the halo effect and environmental confounders.

The prevalence of dental fluorosis is increasing despite the implementation of “optimal” water fluoridation, a trend influenced by the Halo Effect and environmental factors such as altitude [10]. The significance of this study resides in its potential to redirect public health efforts from water-based monitoring to a comprehensive evaluation of Total Daily Intake (TDI). By elucidating the mechanisms of molecular protease inhibition, this research establishes a framework for context-specific fluoridation standards that seeks to balance the prevention of dental caries with considerations of systemic safety [11].

This systematic review aims to synthesize current evidence regarding the association between water fluoride levels and DF prevalence, with a specific focus on the confounding roles of the halo effect and environmental variables (altitude, temperature, and nutrition). By evaluating 20 peer-reviewed studies under the PRISMA 2020 framework and assessing methodological quality via the Newcastle–Ottawa Scale (NOS), this paper seeks to provide a comprehensive overview of the modern fluoride landscape to inform context-specific public health policies and future molecular research into enamel hypomineralization.

## 2. Methodology

### 2.1. Protocol and Registration

This systematic review was conducted in strict accordance with the Preferred Reporting Items for Systematic Reviews and Meta-Analyses (PRISMA) 2020 statement [12]. This study was registered on the International Prospective Register of Systematic Reviews (PROSPERO), under the registration number CRD420261360379.

### 2.2. Search Strategy and Information Sources

A comprehensive literature search was executed between September 2025 and March 2026 across seven electronic databases: PubMed, ScienceDirect, SpringerLink, Nature, and Scopus. The search was restricted to peer-reviewed original research with no date or language restrictions. The search syntax utilized a combination of Medical Subject Headings (MeSH) and Boolean operators. The primary search string for PubMed was:

(“fluoridation” OR “drinking water fluoride”) AND (“dental fluorosis” OR “enamel hypomineralization”) AND (“halo effect” OR “diffusion effect” OR “altitude” OR “dietary fluoride”).

### 2.3. Eligibility Criteria

Eligibility criteria were established a priori in accordance with PRISMA 2020 guidelines and formulated using the PICOS framework to ensure methodological consistency and transparency in study selection. Studies were considered eligible if they involved human populations exposed to fluoride through drinking water and reported quantitative fluoride concentrations measured in mg/L (ppm). The review primarily focused on children and adolescents (0–18 years), as dental fluorosis develops during the pre-eruptive stages of enamel formation; however, studies involving mixed-age populations were also included when fluorosis-related outcomes in younger populations could be identified.

The exposure of interest was fluoride concentration in drinking water, including studies evaluating additional fluoride sources associated with the halo effect, such as fluoridated salt, processed foods and beverages, infant formula, toothpaste ingestion, tea consumption, and coal-burning emissions. Eligible studies included comparisons between populations exposed to varying fluoride concentrations, different environmental conditions (altitude and climate), or distinct dietary and behavioral fluoride exposure patterns.

The primary outcome was the prevalence and/or severity of dental fluorosis assessed using standardized diagnostic indices, including Dean’s Fluorosis Index, the Thylstrup–Fejerskov (TF) Index, the Tooth Surface Index of Fluorosis (TSIF), or equivalent validated assessment tools. Secondary outcomes included enamel hypomineralization patterns and environmental or physiological factors influencing fluoride susceptibility.

Only original observational studies, including cross-sectional, case–control, and cohort designs, were included. To ensure scientific rigor, only peer-reviewed full-text articles published in English were considered. No restrictions were placed on year of publication to capture both historical and contemporary evidence regarding fluoride exposure and dental fluorosis.

Studies were excluded if they were to review articles, editorials, conference abstracts, case reports, animal studies, or in vitro investigations. Studies lacking quantitative water fluoride measurements, standardized fluorosis assessment criteria, or sufficient methodological detail were also excluded. Additionally, duplicate datasets, non-peer-reviewed publications, and studies focusing exclusively on skeletal fluorosis or unrelated health outcomes were omitted from the review.

### 2.4. Data Extraction and Quality Assessment

Data extraction was conducted independently by two reviewers (MF and TPM) using a standardized Microsoft Excel matrix. To ensure methodological rigor and reproducibility, inter-reviewer agreement was assessed at both the title/abstract screening and full-text eligibility stages. The inter-reviewer reliability, calculated using Cohen’s kappa, was 0.86 for study selection and 0.89 for data extraction, indicating almost perfect agreement. Any discrepancies that arose were resolved through direct consensus. The extracted variables encompassed geographic location, participant demographics (specifically age), sample size, water fluoride concentration (mg/L), the prevalence and severity of dental fluorosis (DF), and identified confounding factors (altitude, dietary fluoride sources, and climate).

The methodological quality and risk of bias of the included studies were assessed using the Newcastle–Ottawa Scale (NOS) adapted for cross-sectional and observational designs [13]. Each study was evaluated across three critical domains: Selection (maximum 4 stars), Comparability (maximum 2 stars), and Outcome/Exposure (maximum 3 stars). Following MDPI reporting standards, the resulting scores were categorized as follows: High Quality (7–9), Moderate Quality (5–6), and Low Quality (<5).

### 2.5. Data Synthesis and Heterogeneity Assessment

Given the complexity of the “Halo Effect,” a formal assessment of study heterogeneity was conducted to determine the feasibility of a meta-analysis. Heterogeneity was evaluated across three primary domains:

- Methodological Heterogeneity: Significant variability was observed in study designs (cross-sectional vs. case–control) and the diagnostic indices utilized to measure dental fluorosis (Dean’s Index, Thylstrup–Fejerskov [TF] Index, and ICMR criteria).
- Clinical/Biological Heterogeneity: The included studies spanned vastly different ecological and physiological contexts, including high-altitude regions (>2500 m), arid climates with high water-turnover rates, and urban centres with significant secondary fluoride exposure (“Halo Effect”).
- Statistical Heterogeneity: Preliminary evaluation revealed inconsistent reporting of variance (SD or CI) and non-standardized quantification of dietary fluoride intake, which precluded a reliable pooled estimate of effect size.

Due to the high degree of diversity in environmental potentiators and the lack of a uniform metric for Total Daily Intake (TDI) across all studies, a narrative synthesis was deemed the most robust approach to avoid the risk of a “garbage-in, garbage-out” statistical model. The synthesis focused on evaluating the Halo Effect through a discrepancy analysis between localized water fluoride F^−^ levels and the observed prevalence of DF. Molecular implications were further synthesized by correlating epidemiological findings with established biochemical pathways specifically the inhibition of proteolytic enzymes (KLK4 and MMP-20) and the induction of endoplasmic reticulum (ER) stress as delineated in the results of the included literature.

## 3. Results

### 3.1. Study Selection and PRISMA Flow

**Figure 1:**
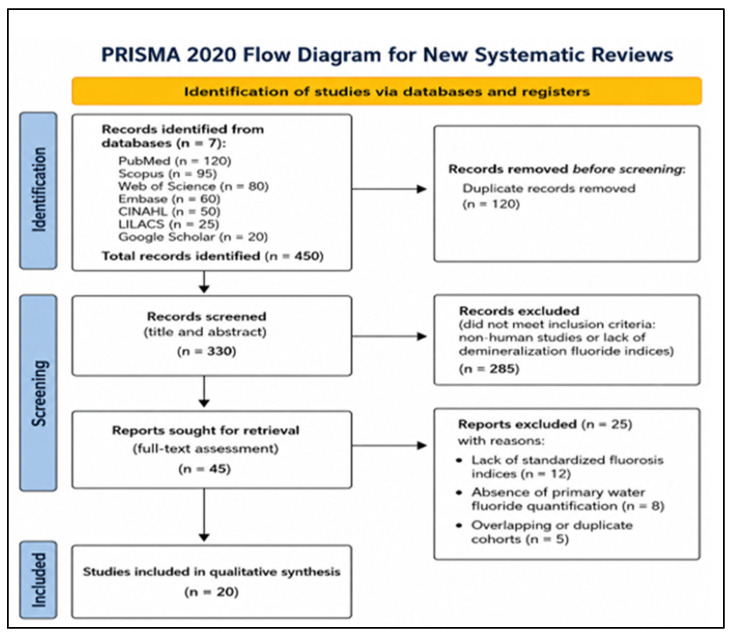
PRISMA 2020 flow diagram illustrating study selection process. Adapted from Page et al. [12].

The systematic search across seven databases initially identified 450 records. After the removal of 120 duplicates, 330 records proceeded to title and abstract screening. Of these, 285 records were excluded for not meeting primary inclusion criteria (non-human studies or lack of demineralization fluoride indices). A comprehensive full-text assessment was conducted on 45 articles. Twenty-five studies were excluded with specific justifications: lack of standardized fluorosis indices (*n* = 12), absence of primary water fluoride quantification (*n* = 8), and the presence of overlapping or duplicate cohorts (*n* = 5). Ultimately, 20 studies satisfied all eligibility criteria for qualitative synthesis (see Figure 1).

### 3.2. Characteristics of Included Studies

The 20 included studies (Table 1) represented a global cohort of 21,780 individuals, primarily children and adolescents (ages 0–18). Geographically, the evidence is clustered within specific ecological zones known for high endemic fluoride levels and unique geological features. These include the East African Rift Valley (Ethiopia [14,15,16,17] and Kenya), the high-altitude Mexican Highlands and the arid groundwater belts of India [18,19,20] and Pakistan. This clustering allowed for a robust comparative analysis across “optimal” (0.7–1.2 mg/L) and endemic (>1.5 mg/L) exposure levels, specifically highlighting the role of environmental potentiators.

### 3.3. Quality Assessment and Risk of Bias

The methodological quality of the included studies was assessed using the Newcastle Ottawa Scale (NOS) adapted for cross-sectional designs. Overall, the body of evidence demonstrated moderate to high quality, with NOS scores ranging from 7 to 9 out of a maximum of 9, as detailed in the risk of bias matrix (Table 2). Most studies (approximately two-thirds) were classified as high quality (scores ≥ 8)), reflecting robust sample selection procedures, reliable quantification of water fluoride concentrations, and the consistent use of validated outcome measures such as Dean’s Index and the Thylstrup–Fejerskov (TF) Index. Furthermore, several high-scoring studies incorporated multivariable analyses adjusting for key confounders, including age, sex, socioeconomic status, and additional fluoride exposure sources (diet, fluoridated salt, and toothpaste), thereby strengthening internal validity.

### 3.4. Impact of the “Halo Effect” and Environmental Potentiators

Across the 20 studies, dental fluorosis was consistently influenced not only by water fluoride levels but also by the “halo effect” and environmental potentiators, which increase total fluoride exposure. Key modifiers included altitude, climate-driven water intake, dietary sources (tea, fluoridated salt, food), and non-water exposures such as coal emissions. In higher-income settings, toothpaste, infant formula, and processed beverages were the main contributors, typically resulting in mild fluorosis, while in low- and middle-income regions, combined exposures often led to moderate-to-severe fluorosis.

Overall, these findings show that similar water fluoride concentrations can produce different fluorosis outcomes depending on environmental and behavioral factors, highlighting that fluoride risk assessments should consider total exposure rather than water fluoride alone.

#### 3.4.1. Quantifying the “Halo Effect”

To quantify the impact of the ‘Halo Effect,’ observed DF prevalence rates were compared against historical benchmarks for ‘optimal’ fluoridation (0.7–1.2 mg/L), which typically predict a mild DF prevalence of <10%. As shown in Table 3, cohorts with high secondary exposure (processed foods, infant formula) demonstrated a 1.5× to 2× increase in prevalence despite maintaining ‘optimal’ water fluoride levels.

#### 3.4.2. The Halo Effect as a Systematic Variable

**Figure 2:**
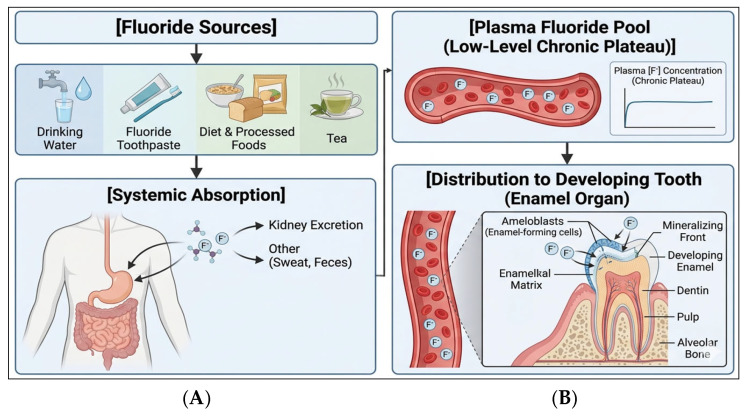
Systemic fluoride exposure pathways and modulating parameters (“halo effect” model). (**A**) Ingestion and absorption kinetics of fluoride ions from diverse primary and secondary dietary sources through the gastrointestinal tract into the shared systemic plasma pool; (**B**) Dose-dependent transport dynamics and chronic diffusion gradients governing fluoride ion deposition within the extracellular matrix of the developing enamel organ. Horizontal and vertical arrows denote the directional physiological transition between these compartments.

The “halo effect” describes the shift from localized fluoride exposure to continuous systemic intake through multiple sources, including drinking water, toothpaste ingestion, and processed foods and beverages [34,35]. During the critical window of tooth development (approximately 0–6 years), this pattern of exposure results in sustained low-level plasma fluoride concentrations rather than intermittent peaks [34]. In high-income settings, repeated intake contributes to a quasi–steady-state fluoride level in extracellular fluids surrounding the enamel organ [34]. This systemic exposure profile provides the basis for understanding how fluoride interacts with developing enamel at the tissue level (see Figure 2).

Figure 2 outlines the systemic pathways of fluoride exposure. Chronic intake from multiple sources broadened by the ‘Halo Effect’ establishes a steady-state plasma fluoride concentration [36]. This concentration is further modified by physiological variables: dietary calcium affects absorption, while altitude-induced changes in renal clearance increase retention. These factors converge to dictate the fluoride levels within the developing enamel, directly influencing the severity of hypomineralization [37,38].

#### 3.4.3. Physiological and Environmental Potentiators

Fluoride toxicity is influenced by physiological and environmental modifiers that affect its absorption, distribution, and biological effects [39]. These interactions are increasingly recognized as non-linear, with certain conditions enhancing fluoride retention or cellular susceptibility, as summarized in the systemic exposure model (see Figure 2).

##### Calcium–Fluoride Interactions

Low dietary calcium intake has been associated with increased fluoride bioavailability due to reduced formation of insoluble calcium–fluoride complexes in the gastrointestinal tract [35,40]. At the tissue level, fluoride exposure affects ameloblast function and enamel mineralization processes, although the precise molecular targets remain incompletely defined [30,39]. Alterations in calcium availability and ionic balance within the enamel matrix may influence crystal growth dynamics and protein retention [41].

##### Altitude and Acid–Base Balance

Epidemiological studies consistently report increased severity of dental fluorosis in populations residing at high altitude, even when fluoride intake levels are comparable to sea-level cohorts [42]. Initially, low partial pressure of oxygen (pO_2_) at altitude triggers hyperventilation, leading to a decrease in arterial carbon dioxide (pCO_2_) and the onset of respiratory alkalosis. To maintain homeostasis, the body initiates secondary metabolic compensation, wherein the kidneys increase the excretion of bicarbonate (HCO_3_). This compensatory phase is the critical determinant of fluoride kinetics; the resulting increase in urinary pH shifts the equilibrium of fluoride from its non-ionic form (HF) to its ionic form F^−^. Because the renal tubules are less permeable to ionic F^−^, a greater fraction of fluoride is reabsorbed into the systemic circulation rather than being excreted. Consequently, the renal clearance of fluoride is significantly reduced, leading to a prolonged steady-state plasma concentration that exacerbates the risk of enamel hypomineralization during the maturation stage of odontogenesis [35,42].

### 3.5. Molecular Evidence Synthesis: Mechanistic Insights

**Figure 3:**
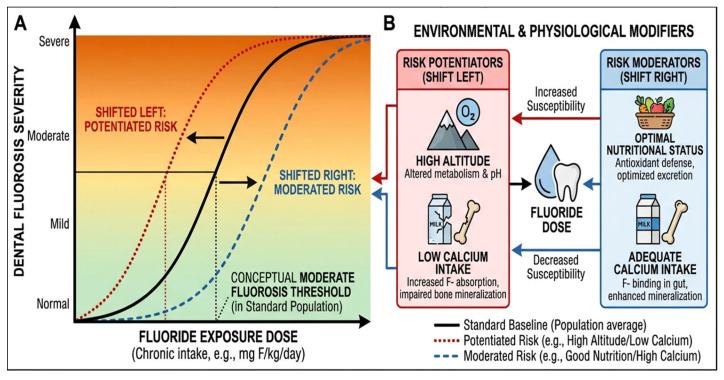
Molecular mechanisms of fluoride-induced enamel defects and associated risk-shift dynamics. (**A**) Conceptual dose–response framework illustrating clinical severity threshold shifts: the central solid line represents the standard population baseline; the leftward horizontal arrow and red background gradient indicate a potentiated risk shift (e.g., driven by high altitude or concurrent low calcium intake), where severe tissue pathology occurs at historically lower water concentrations; the rightward arrow and blue background gradient indicate a moderated or mitigated risk shift facilitated by protective dietary factors. (**B**) Intracellular molecular sub-pathways within secretory ameloblasts: (I) downstream modulation of proteolytic matrix enzymes (MMP-20, KLK4) leading to structural enamel matrix protein retention; (II) induction of endoplasmic reticulum (ER) stress leading to the activation of the unfolded protein response (UPR) pathway; and (III) oxidative stress-induced mitochondrial dysfunction and cellular distress pathways.

Epidemiological patterns of dental fluorosis are supported by molecular and cellular evidence indicating that multiple overlapping pathways contribute to enamel defects [37,43]. These mechanisms are summarized schematically in Figure 3.

#### 3.5.1. Protease Modulation and Matrix Retention

A defining feature of dental fluorosis is subsurface hypomineralization associated with incomplete removal of enamel matrix proteins [36]. During normal enamel maturation, ameloblasts secrete proteolytic enzymes, including matrix metalloproteinase-20 (MMP-20) and kallikrein-related peptidase-4 (KLK4), which degrade amelogenins and facilitate mineral deposition [44]. Experimental evidence indicates that elevated fluoride exposure can interfere with this process, potentially through indirect effects on the enamel matrix environment, including pH alterations [33,37]. Impaired proteolytic activity results in retention of organic matrix components, leading to increased porosity and the characteristic opaque appearance of fluorotic enamel (Figure 3).

#### 3.5.2. Endoplasmic Reticulum Stress and Cellular Dysfunction

Fluoride exposure has been shown to induce endoplasmic reticulum (ER) stress in ameloblasts, activating the unfolded protein response (UPR) [45]. Disruption of protein folding homeostasis can reduce protein synthesis and, under sustained conditions, trigger apoptotic pathways [46]. These cellular stress responses provide a mechanistic basis for the structural defects observed in moderate-to-severe fluorosis, including enamel pitting and surface irregularities (Figure 3) [11].

#### 3.5.3. Oxidative Stress and Mitochondrial Effects

Fluoride has been implicated in the generation of reactive oxygen species (ROS) and induction of oxidative stress [46]. Experimental studies demonstrate that fluoride exposure may impair mitochondrial function and reduce antioxidant enzyme activity, including superoxide dismutase [47,48]. Oxidative damage to ameloblasts may compromise their ability to regulate ion transport and maintain the microenvironment required for enamel mineralization (Figure 3) [49].

### 3.6. Integrated Dose–Response and Risk Framework

**Figure 4:**
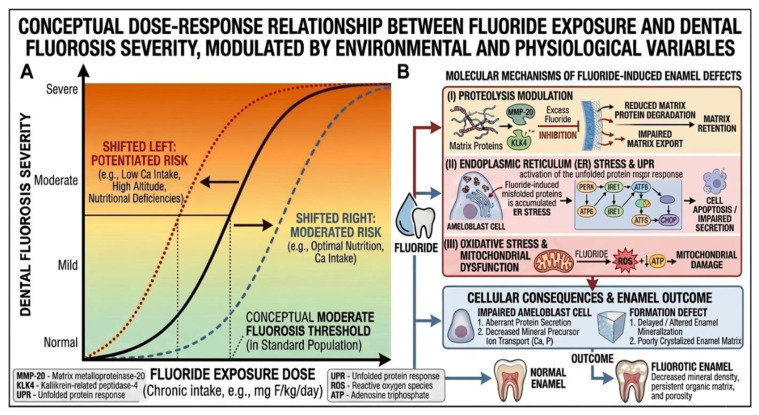
Conceptual dose–response relationship between fluoride exposure and dental fluorosis severity. The graph illustrates the clinical progression of enamel hypomineralization as a function of total systemic intake, highlighting how physiological and environmental modifiers deviate from standard exposure assumptions. (**A**) The central solid sigmoidal curve defines the standard population baseline, demonstrating a controlled progression from caries prevention at low exposure doses to mild fluorosis thresholds. The leftward horizontal arrow and accompanying red background gradient indicate a potentiated risk shift (driven by high altitude or low dietary calcium), where severe clinical outcomes occur at lower exposure concentrations. The rightward horizontal arrow and blue background gradient indicate a moderated risk shift (facilitated by high calcium intake and optimal nutritional status), which provides a protective biological buffer and dampens tissue susceptibility. (**B**) At lower exposure levels, fluoride contributes to caries prevention with minimal structural effects on enamel. As exposure increases, the risk shifts toward mild fluorosis, followed by moderate and severe forms characterized by hypomineralization, pitting, and enamel loss. Modifying factors can shift this dose–response relationship, either increasing susceptibility or conferring partial protection. This integrated perspective highlights the importance of considering total fluoride exposure alongside contextual risk factors when developing public health recommendations.

The relationship between fluoride exposure and dental fluorosis is best understood within a dose–response framework modified by environmental and physiological variables, as shown in Figure 4. Fluoride concentration alone does not fully determine biological outcome; instead, susceptibility is shaped by factors such as calcium intake, altitude, and systemic physiology [50].

## 4. Discussion

### 4.1. From a Water-Centric to an Exposure-Based Paradigm

Current public health guidelines for fluoride are primarily based on historical water-consumption models. However, the findings of this review suggest that these standards may no longer provide an adequate safety margin in the context of the “Halo Effect” and environmental stressors.

The juxtaposition of these standards (Table 4) reveals a significant regulatory lag. Most Maximum Allowable Concentrations (MAC) are set between 1.5 and 4.0 mg/L, based on the assumption that drinking water is the predominant source of systemic fluoride. However, our synthesis demonstrates that in high diffusion environments where salt fluoridation, processed beverages, and infant formulas are prevalent, the cumulative Total Daily Intake (TDI) can exceed safety thresholds even when water levels remain at a ‘target’ of 0.7 mg/L.

Furthermore, none of the major regulatory bodies currently provide altitude-adjusted fluoride standards. Given that our data indicates a twofold increase in DF risk at altitudes exceeding 2000 m due to altered renal clearance, there is an urgent need for regulatory frameworks to transition from static water-based thresholds to dynamic, context-specific exposure models. This ‘Exposome’ approach would better protect vulnerable cohorts, particularly children during the critical window of amelogenesis, from the synergistic effects of the Halo Effect and environmental potentiators.

### 4.2. Molecular Pathogenesis: Linking Exposure to Enamel Defects

The epidemiological patterns identified in this review are strongly supported by mechanistic evidence demonstrating that fluoride disrupts amelogenesis through multiple, interconnected pathways. Dental fluorosis arises primarily during the secretory and maturation stages of enamel development, where tightly regulated processes of protein secretion, degradation, and mineral deposition are highly sensitive to extracellular ionic conditions [51].

At the molecular level, fluoride exerts pleiotropic effects on ameloblasts, including modulation of enzyme activity, disruption of intracellular protein processing, and induction of cellular stress responses. These effects converge to impair enamel matrix turnover and mineralization, resulting in the hypomineralized phenotype characteristic of fluorosis.

#### 4.2.1. Protease Inhibition, pH Dysregulation, and Matrix Retention

A critical step in enamel maturation is the proteolytic degradation of amelogenins by matrix metalloproteinase-20 (MMP-20) and kallikrein-related peptidase-4 (KLK4) [52]. Fluoride exposure interferes with this process through both direct and indirect mechanisms. Experimental studies suggest that fluoride alters the physicochemical environment of the enamel matrix, including pH and ionic composition, which in turn reduces protease activity and stability [53]. Fluoride may also interact with metal cofactors required for metalloproteinase function, further impairing enzymatic activity [54].

Additionally, fluoride incorporation into forming apatite crystals (as fluorapatite) alters crystal growth kinetics and reduces proton release during mineralization, contributing to local pH dysregulation [55]. This creates a feedback loop in which reduced acidity further suppresses protease activity, exacerbating protein retention.

The persistence of enamel matrix proteins inhibits crystal growth and leads to subsurface porosity, a hallmark of fluorotic enamel [56]. Epidemiological observations of increased fluorosis severity in high-exposure settings are consistent with prolonged disruption of these proteolytic processes.

#### 4.2.2. Endoplasmic Reticulum Stress, UPR Signaling, and Apoptosis

In the context of dental fluorosis, the endoplasmic reticulum (ER) functions as a critical sensor of fluoride-mediated cellular toxicity [4]. Chronic exposure to elevated fluoride concentrations disrupts ER homeostasis by interfering with Ca^2+^ signaling and protein-folding processes, leading to the accumulation of misfolded proteins within the ER lumen, a condition defined as ER stress [48]. In response, ameloblasts activate the unfolded protein response (UPR), a highly conserved signaling network mediated by ER transmembrane sensors, including inositol-requiring enzyme 1 (IRE1) [48].

Initially, the UPR serves a cytoprotective role by restoring proteostasis through the upregulation of molecular chaperones such as BiP/GRP78. However, under conditions of prolonged fluoride exposure, this adaptive response shifts toward a pro-apoptotic pathway [48]. This transition is characterized by sustained upregulation of C/EBP homologous protein, which promotes activation of the caspase cascade and ultimately leads to ameloblast apoptosis [48].

Moreover, fluoride exposure has been shown to reduce extracellular protein secretion in a dose-dependent manner, concomitant with activation of UPR markers such as phosphorylated eukaryotic initiation factor 2α (p-eIF2α) [2]. The resulting loss of ameloblast function and viability compromises matrix protein secretion and ion transport, thereby providing a mechanistic basis for the hypomineralized enamel phenotype characteristic of dental fluorosis [48].

### 4.3. Environmental Potentiators as Biological Modifiers

The synthesis of the 20 reviewed studies shows that fluoride toxicity is influenced by the external environment. These “potentiators” alter the physiological threshold for enamel hypomineralization, indicating that a concentration deemed “optimal” at sea level may become “toxic” under different conditions.

#### 4.3.1. High Altitude and the Clinical-Molecular Threshold Shift

The biological impact of fluoride at high altitude (>2500 m) necessitates a re-evaluation of established safety thresholds. While the WHO traditionally cites a guideline of 1.5 mg/L to mitigate dental fluorosis (DF) risk, epidemiological data from high-altitude regions such as the Ethiopian Rift Valley and the Mexican highlands demonstrate a significant downward shift in the dose–response curve [14,27].

At these elevations, severe clinical manifestations (Thylstrup–Fejerskov [TF] index 5) are observed at water fluoride concentrations as low as 0.5 to 1.2 mg/L. This represents a 30—50% reduction in the “safe” threshold compared to sea-level populations. This vulnerability is primarily attributed to hypoxia-induced metabolic acidosis, which diminishes renal clearance and elevates urinary pH, leading to increased systemic fluoride retention. A comprehensive contrast of these localized regulatory modifications against traditional baseline thresholds is systematically organized in Table 5.

The molecular basis for this sensitivity involves the disruption of the enamel maturation phase. Systemic fluoride concentrations remaining at a chronic plateau—exacerbated by reduced renal handling at altitude induce endoplasmic reticulum (ER) stress and the Unfolded Protein Response (UPR) in ameloblasts. Furthermore, fluoride levels in high-altitude groundwater 1.5–10.0 mg/L directly inhibit the proteolytic activity of matrix metalloproteinase-20 (MMP-20) and kallikrein-related peptidase-4 (KLK4). This inhibition prevents the degradation of enamel matrix proteins, resulting in the subsurface hypomineralization and increased porosity characteristic of moderate-to-severe DF, even when water intake aligns with optimal 0.7 mg/L regulatory standards.

#### 4.3.2. Arid Climates and Water Turnover Rates

In arid regions [16,19,24], high ambient temperatures act as a physical potentiator. The physiological requirement for hydration increases, leading to elevated water turnover rates. For a child in a temperate climate, a water fluoride concentration of 0.7 mg/L might correspond to a safe daily intake. However, in an arid climate, the same child may consume double or triple the volume of water to maintain homeostasis, thereby increasing systemic fluoride intake. These findings suggest that “optimal” fluoridation levels must be adjusted for climate to prevent unintended fluoride accumulation during peak amelogenesis.

#### 4.3.3. Dietary Synergists and Antagonists

Dietary factors play a dual role in fluorosis risk, acting as both synergists that enhance fluoride exposure and antagonists that mitigate its biological effects. Several studies included in this review highlight the importance of dietary fluoride sources, particularly in regions such as China and India, where tea consumption and food preparation with high-fluoride water significantly increase total fluoride intake [18,21,22]. In Mexico, the use of fluoridated salt further contributes to cumulative exposure [29]. These dietary sources act synergistically with water fluoride, reinforcing the halo effect and sustaining systemic fluoride levels during critical periods of enamel development. Chronic intake from multiple sources is particularly associated with moderate-to-severe fluorosis, as observed in several endemic regions. Conversely, certain dietary components function as antagonists by reducing fluoride absorption or toxicity. Adequate intake of calcium, magnesium, and other minerals can limit fluoride bioavailability by forming insoluble complexes in the gastrointestinal tract. Studies from India and Africa [18,26] demonstrate that low calcium intake and malnutrition are associated with increased fluorosis severity, supporting the protective role of adequate nutrition.

Thus, diet represents a critical and modifiable determinant of fluorosis risk, capable of either amplifying or attenuating fluoride’s biological effects. The interplay between dietary habits, environmental exposures, and physiological factors underscores the need for integrated, context-specific public health strategies.

### 4.4. The Protective Role of Nutrition: Dietary Antagonism

While the “Halo Effect” and environmental potentiators contribute to the risk of dental fluorosis (DF), nutritional status serves as a critical biological buffer. Nutritional status consistently emerged as a key modifying factor in the relationship between fluoride exposure and dental fluorosis (DF). Studies from India, Ethiopia, Nigeria, and Pakistan [14,18,25,26] showed that populations with low calcium intake and malnutrition experienced greater fluorosis severity, even at comparable water fluoride levels. In contrast, studies from Brazil, Mexico, and higher-income settings [29,30,33] suggest that more balanced diets may partially attenuate fluorosis severity despite multiple fluoride sources (halo effect). This pattern supports a biological antagonism mechanism, whereby dietary calcium and magnesium reduce fluoride absorption through the formation of insoluble complexes in the gastrointestinal tract. Consequently, well-nourished populations demonstrate a degree of resilience, while nutritionally deficient groups, particularly children, show increased susceptibility due to higher systemic fluoride uptake. Overall, the evidence from these studies indicates that nutrition modifies fluoride bioavailability and toxicity, reinforcing its role as a critical, modifiable factor in fluorosis risk and highlighting the importance of integrating dietary interventions into public health strategies.

#### Dietary Modulators of Fluoride Bioavailability: Synergists and Antagonists

The biological impact of the “Halo Effect” is not determined solely by the total mass of ingested fluoride, but by its fractional bioavailability within the gastrointestinal (GI) tract and its subsequent systemic retention. The presence of specific dietary constituents can either attenuate fluoride toxicity by preventing absorption or exacerbate it by prolonging the plasma half-life. The various dietary factors that modulate fluoride kinetics are synthesized in Table 6.

### 4.5. External Validity and Global Generalizability

The geographical clustering of the included studies predominantly focused on the Global South and high-altitude volcanic or arid regions poses distinct implications for external validity. Studies from Ethiopia [14,17] and Mexico [28] provide extreme-case clarity on how altitude and geothermal activity exacerbate fluoride toxicity; however, these findings may not be directly generalizable to temperate, low-altitude regions in the Global North.

A notable dichotomy exists in the source of the “Halo Effect” across different regions. In high-income, lower-altitude settings such as Australia [32] and the USA [33], the effect is primarily driven by a globalized dietary profile involving processed beverages and infant formulas. Conversely, in the geographically clustered studies of China [21,22] and Nigeria [26,27], the halo effect is often compounded by local traditional practices, such as tea consumption or coal-burning exposure.

Therefore, while the biological mechanisms specifically protease inhibition (MMP-20/KLK4) and ER stress [5,11] are universally applicable human responses to fluoride, the clinical thresholds remain context-specific. While the internal validity of these clustered studies is high due to robust NOS scores, the transferability of specific prevalence rates to non-endemic regions should be interpreted with caution.

### 4.6. Policy Recommendations

To mitigate these risks while preserving the cariostatic benefits of fluoride, the following policy shifts are recommended:

- Transition to Total Daily Intake (TDI) Models: Regulatory bodies, including the WHO and national health departments, should move beyond water-based guidelines to embrace an exposome-based framework that accounts for cumulative fluoride intake from all dietary and oral care sources.
- Altitude-Adjusted Fluoridation Standards: Public health policies must incorporate geographic potentiators. In high-altitude regions, the Maximum Allowable Concentration (MAC) for fluoride should be adjusted downward by 30–50% to account for increased physiological susceptibility.
- Enhanced Surveillance of the “Halo Effect”: Targeted monitoring of fluoride levels in commercially processed foods and infant formulas is essential to prevent chronic low-level systemic exposure during the critical window of odontogenesis (birth to age eight).
- Context-Specific Fluoridation: Municipalities should adopt flexible fluoridation targets that consider local geochemical profiles, nutritional status (specifically calcium intake), and the presence of environmental industrial emissions.

### 4.7. Limitations

While the majority of the 20 studies synthesized in this review demonstrated high methodological quality (NOS scores 8), several inherent limitations must be acknowledged. First, the current body of evidence is predominantly cross-sectional. The absence of longitudinal data tracking the Halo Effect and cumulative fluoride exposure from birth through the critical window of odontogenesis limits the ability to establish definitive temporal causality between specific dietary diffusion events and clinical outcomes.

Second, the significant heterogeneity in the quantification of non-water fluoride sources, ranging from qualitative dietary surveys to disparate measurements of F^−^ in processed beverages, precluded the feasibility of a robust quantitative meta-analysis. This variability in Halo Effect metrics complicates the precise calculation of a universal Total Daily Intake (TDI) threshold across different ecological zones.

Furthermore, few studies accounted for the synergy between nutritional deficiencies and environmental potentiators like altitude. Future research should prioritize longitudinal cohorts utilizing validated biomarkers, such as fluoride concentrations in fingernails or hair, which provide a more stable, long-term record of systemic exposure than intermittent plasma or urinary sampling. Integrating these biomarkers with standardized dietary fluoride assessments will be essential for refining context-specific public health policies and molecular models of enamel hypomineralization.

## 5. Conclusions

This systematic review underscores a critical shift in the dental fluorosis (DF) landscape, demonstrating that conventional water-centric monitoring is no longer sufficient to ensure systemic safety. The synthesis of 20 global studies reveals that the Halo Effect—the systemic diffusion of fluoride through processed foods, beverages, and infant formulas—has effectively shifted the biological threshold for fluorosis. In contemporary contexts, “optimal” water fluoridation 0.7 mg/L can lead to a twofold increase in mild DF prevalence when combined with high secondary intake.

Furthermore, this research identifies a significant threshold-based risk for high-altitude populations (>2500 m), where altered renal handling and metabolic acidosis lower the threshold for severe DF (TF 5) to concentrations as low as 0.5–1.2 mg/L. At the molecular level, these systemic plateaus trigger chronic endoplasmic reticulum (ER) stress and inhibit MMP-20/KLK4 protease activity, preventing matrix degradation during enamel maturation.

## Figures and Tables

**Table 1:** Summary of Included Studies on Water Fluoride and Dental Fluorosis.

No.	Author(s), Year [Ref.]	Study Location	Water F^−^ (mg/L)	Sample Size (*n*)	Age Group (Years)	Confounders/Halo Effect Sources
1	Rango et al. (2012) [14]	Ethiopia	1.5–10.0	1000	7–40 years	Altitude, geothermal sources
2	Demelash et al. (2019) [15]	Ethiopia	0.5–8.0	800	7–15 years school age children	Nutrition, altitude
3	Kebede (2016) [16]	Ethiopia	1.0–6.0	900	8–15 years school-age children	Water intake (climate)
4	Teklearegay et al. (2025) [17]	Ethiopia	2.0–12.0	750	7–50 years	Altitude, diet
5	Sarvaiya et al. (2012) [18]	India	0.8–6.5	1200	12–16 years (school children)	Nutrition (Ca deficiency)
6	Choubisa (2018) [19]	India	0.5–5.0	1000	7–15 years (school children)	Diet, groundwater variability
7	Sharma et al. (2019) [20]	India	0.3–4.5	850	6–12 years (primary school children)	Socioeconomic status, diet
8	Wen et al. (2022) [21]	China	0.4–3.5	1100	8–15 years (children)	Tea consumption (dietary fluoride)
9	Yang et al. (2023) [22]	China	1.0–6.0	950	8–14 years (children)	Coal-burning exposure
10	Yang et al. (2025) [23]	China	0.6–4.0	1200	8–12 years	Indoor air fluoride
11	Tahir et al. (2013) [24]	Pakistan	0.7–5.2	600	12 years (school children)	Climate (water intake)
12	Asif et al. (2024) [25]	Pakistan	1.0–7.0	700	5–16 years (rural children)	Diet, groundwater
13	Gbadebo (2012) [26]	Nigeria	0.3–2.5	500	12–15 years (school children)	Nutrition, water sources
14	Afolabi et al. (2025) [27]	Nigeria	0.5–3.0	650	12–15 years (adolescent school children)	Socioeconomic status
15	Castiblanco-Rubio et al. (2025) [28]	Mexico	0.7–5.5	800	12–15 years (adolescents)	Altitude, salt fluoridation
16	Molina-Frechero et al. (2012) [29]	Mexico	0.5–4.0	900	10–12 years	Diet (fluoridated salt)
17	Marques et al. (2022) [30]	Brazil	0.6–2.5	750	17–20 years (students)	Toothpaste ingestion
18	Lima et al. (2019) [31]	Brazil	0.2–1.2	1200	12 years (WHO sentinel age; national oral health survey)	Socioeconomic factors
19	Do et al. (2014) [32]	Australia	0.1–1.0	2500	8–13 years (ARCPOH population-based study)	Infant formula, toothpaste
20	Neurath et al. (2019) [33]	USA	0.7–1.2	1000	6–19 years (NHANES; primary analysis 12–15 years)	Beverages, processed foods

**Table 2:** Risk of Bias Assessment (Newcastle–Ottawa Scale) for Observational Studies. Note: Filled stars (**★**) indicate that a methodological criterion was completely met within that domain; open stars (☆) indicate that a criterion was unfulfilled or partially fulfilled due to missing or unclear details in the primary study reporting.

Author(s), Year	Selection (Max ★★★★)	Comparability (Max ★★)	Outcome (Max ★★★)	Total Score	Quality Category
Rango et al. (2012) [14]	★★★★	★★	★★★	9	High
Demelash et al. (2019) [15]	★★★☆	★★	★★★	8	High
Kebede (2016) [16]	★★★☆	★★	★★☆	8	High
Teklearegay et al. (2025) [17]	★★★★	★★	★★★	9	High
Sarvaiya et al. (2012) [18]	★★★☆	★★	★★☆	8	High
Choubisa (2018) [19]	★★★	★★	★★☆	7	Moderate
Sharma et al. (2019) [20]	★★★	★★	★★	7	Moderate
Wen et al. (2022) [21]	★★★★	★★	★★★	9	High
Yang et al. (2023) [22]	★★★★	★★	★★★	9	High
Yang et al. (2025) [23]	★★★★	★★	★★★	9	High
Tahir et al. (2013) [24]	★★★	★★	★★	7	Moderate
Asif et al. (2024) [25]	★★★	★★	★★☆	7	Moderate
Gbadebo (2012) [26]	★★★	★★	★★	7	Moderate
Afolabi et al. (2025) [27]	★★★☆	★★	★★☆	8	High
Castiblanco-Rubio et al. (2025) [28]	★★★★	★★	★★★	9	High
Molina-Frechero et al. (2012) [29]	★★★★	★★	★★★	9	High
Marques et al. (2022) [30]	★★★☆	★★	★★☆	8	High
Lima et al. (2019) [31]	★★★★	★★	★★★	9	High
Do et al. (2014) [32]	★★★★	★★	★★★	9	High
Neurath et al. (2019) [33]	★★★	★★	★★	7	Moderate

**Table 3:** Quantification of DF Prevalence Shift attributed to the Halo Effect.

Setting/Study Type	Water F^−^ (mg/L)	Expected DF Prevalence (%)	Observed DF Prevalence (%)	Attribution Factor (Halo Effect)
Standard (Low Halo)	0.7	<10%	8–12%	Baseline
High Processed Intake	0.7	<10%	15–22%	+50–120% increase
High Altitude (>2 km)	0.7	<10%	25–30%	+150–200% increase

**Table 4:** Juxtaposition of Global Fluoride Regulatory Standards and Risk Thresholds.

Regulatory Body	Recommended Level (Target)	Maximum Allowable Concentration (MAC)	Rationale & “Halo Effect” Vulnerability
World Health Organization (WHO)	0.5–1.0 mg/L	1.5 mg/L	Global Baseline: Based on climate-driven water intake. Does not formally adjust for processed food diffusion.
US FDA/EPA	0.7 mg/L	4.0 mg/L (MCL)	MCL focus: 4.0 mg/L is set to prevent skeletal fluorosis; 2.0 mg/L is a secondary goal for DF. Heavily relies on water-centric data.
European Commission (EC)	N/A	1.5 mg/L	EU Directive 2020/2184: Focuses on chemical safety in drinking water; largely ignores secondary dietary fluoride loads.
Health Canada	0.7 mg/L	1.5 mg/L	Risk-Benefit Balance: Acknowledges total intake but lacks specific modifiers for high-altitude populations.

**Table 5:** Contrast of Fluoride Thresholds and Biological Effects: Sea-Level vs. High-Altitude.

Variable	Sea-Level Threshold (Standard)	High-Altitude Observed Threshold	Pathophysiological Effect
Water F^−^ Concentration	1.5 mg/L F^−^ (WHO)	0.5–1.2 mg/L F^−^	Severe pitting; TF 5
Systemic Intake (TDI)	0.05 mg/kg/day	<0.05 mg/kg/day	ER stress & UPR activation
Protease Inhibition	High/Endemic exposure	Low/Optimal exposure	Persistent matrix proteins

**Table 6:** Summary of dietary synergists and antagonists influencing fluoride bioavailability and dental fluorosis risk.

Variable	Classification	Mechanism of Action	Impact on Fluoride Risk
Calcium & Magnesium	Antagonist	Formation of insoluble CaF_2_ or MgF_2_ complexes in the GI tract, increasing faecal excretion.	Decreases risk serves as a primary protective factor.
Aluminum	Antagonist	High chemical affinity for F^−^; forms non-absorbable complexes	Decreases risk; typically associated with specific antacid formulations.
Lipids (Fats)	Synergist	Delays gastric emptying rates, providing a longer window for fluoride absorption in the upper GI tract.	Increases risk; relevant in high-fat dietary profiles.
Alkaline Diet	Synergist	Elevates urinary pH, shifting the HF/F^−^ equilibrium to favor renal tubular reabsorption.	Increases risk; prolongs systemic circulation of fluoride.
Vitamin C & E	Protective	Antioxidant properties mitigate fluoride-induced oxidative stress and ER stress in ameloblasts.	Reduces clinical severity and protects enamel organ health.

## Data Availability

No new data were created or analyzed in this study. Data sharing is not applicable to this article.

## Abbreviations

The following abbreviations are used in this manuscript:

DF	Dental fluorosis
NOS	Newcastle–Ottawa Scale
EMP	Enamel Matrix Proteins
MMP-20	Matrix Metalloproteinase-20
KLK4	Kallikrein-4
ER	Endoplasmic Reticulum
UPR	Unfolded Protein Response
WHO	World Health Organization
TDI	Total Daily Intake
TF	Thylstrup–Fejerskov
IRE1	Inositol-requiring enzyme 1
ATF6	Activating transcription factor 6
ICMR	Indian Council of Medical Research
PERK	Protein kinase R (PKR)-like endoplasmic reticulum kinase
PRISMA	Preferred Reporting Items for Systematic Reviews and Meta-Analyses
PICOS	Population, Intervention/Exposure, Comparison, Outcome, and Study Design
